# Personality, Gender, and Age in the Language of Social Media: The Open-Vocabulary Approach

**DOI:** 10.1371/journal.pone.0073791

**Published:** 2013-09-25

**Authors:** H. Andrew Schwartz, Johannes C. Eichstaedt, Margaret L. Kern, Lukasz Dziurzynski, Stephanie M. Ramones, Megha Agrawal, Achal Shah, Michal Kosinski, David Stillwell, Martin E. P. Seligman, Lyle H. Ungar

**Affiliations:** 1 Positive Psychology Center, University of Pennsylvania, Philadelphia, Pennsylvania, United States of America; 2 Computer & Information Science, University of Pennsylvania, Philadelphia, Pennsylvania, United States of America; 3 The Psychometrics Centre, University of Cambridge, Cambridge, United Kingdom; University of Warwick, United Kingdom

## Abstract

We analyzed 700 million words, phrases, and topic instances collected from the Facebook messages of 75,000 volunteers, who also took standard personality tests, and found striking variations in language with personality, gender, and age. In our *open-vocabulary* technique, the data itself drives a comprehensive exploration of language that distinguishes people, finding connections that are not captured with traditional closed-vocabulary word-category analyses. Our analyses shed new light on psychosocial processes yielding results that are face valid (e.g., subjects living in high elevations talk about the mountains), tie in with other research (e.g., neurotic people disproportionately use the phrase ‘sick of’ and the word ‘depressed’), suggest new hypotheses (e.g., an active life implies emotional stability), and give detailed insights (males use the possessive ‘my’ when mentioning their ‘wife’ or ‘girlfriend’ more often than females use ‘my’ with ‘husband’ or 'boyfriend’). To date, this represents the largest study, by an order of magnitude, of language and personality.

## Introduction

The social sciences have entered the age of data science, leveraging the unprecedented sources of written language that social media afford [1]–[3]. Through media such as Facebook and Twitter, used regularly by more than 1/7^th^ of the world's population [4], variation in mood has been tracked diurnally and across seasons [5], used to predict the stock market [6], and leveraged to estimate happiness across time [7], [8]. Search patterns on Google detect influenza epidemics weeks before CDC data confirm them [9], and the digitization of books makes possible the quantitative tracking of cultural trends over decades [10]. To make sense of the massive data available, multidisciplinary collaborations between fields such as computational linguistics and the social sciences are needed. Here, we demonstrate an instrument which uniquely describes similarities and differences among groups of people in terms of their differential language use.

Our technique leverages what people say in social media to find distinctive *words*, *phrases*, and *topics* as functions of known attributes of people such as gender, age, location, or psychological characteristics. The standard approach to correlating language use with individual attributes is to examine usage of *a priori* fixed sets of words [11], limiting findings to preconceived relationships with words or categories. In contrast, we extract a data-driven collection of *words*, *phrases*, and *topics*, in which the lexicon is based on the words of the text being analyzed. This yields a comprehensive description of the differences between groups of people for any given attribute, and allows one to find unexpected results. We call approaches like ours, which do not rely on *a priori* word or category judgments, *open-vocabulary* analyses.

We use *differential language analysis* (*DLA*), our particular method of open-vocabulary analysis, to find language features across millions of Facebook messages that distinguish demographic and psychological attributes. From a dataset of over 15.4 million Facebook messages collected from 75 thousand volunteers [12], we extract 700 million instances of *words*, *phrases*, and automatically generated *topics* and correlate them with gender, age, and personality. We replicate traditional language analyses by applying Linguistic Inquiry and Word Count (*LIWC)* [11], a popular tool in psychology, to our data set. Then, we show that *open-vocabulary* analyses can yield additional *insights* (correlations between personality and behavior as manifest through language) and more *information* (as measured through predictive accuracy) than traditional *a priori* word-category approaches. We present a word cloud-based technique to visualize results of *DLA*. Our large set of correlations is made available for others to use (available at: http:www.wwbp.org/).

## Background

This section outlines recent work linking language with personality, gender, and age. In line with the focus of this paper, we predominantly discuss works which sought to gain psychological *insights*. However, we also touch on increasingly popular attempts at *predicting* personality from language in social media, which, for our study, offer an empirical means to compare a *closed vocabulary* analysis (relying on *a priori* word category human judgments) and an *open vocabulary* analysis (not relying on *a priori* word category judgments).

Personality refers to the traits and characteristics that make an individual unique. Although there are multiple ways to classify traits [13], we draw on the popular Five Factor Model (or “Big 5”), which classifies personality traits into five dimensions: *extraversion* (e.g., outgoing, talkative, active), *agreeableness* (e.g., trusting, kind, generous), *conscientiousness* (e.g., self-controlled, responsible, thorough), *neuroticism* (e.g., anxious, depressive, touchy), and *openness* (e.g., intellectual, artistic, insightful) [14]. With work beginning over 50 years ago [15] and journals dedicated to it, the *FFM* is a well-accepted construct of personality [16].

### Automatic Lexical Analysis of Personality, Gender, and Age

By examining what words people use, researchers have long sought a better understanding of human psychology [17]–[19]. As Tauszczik & Pennebaker put it:

Language is the most common and reliable way for people to translate their internal thoughts and emotions into a form that others can understand. Words and language, then, are the very stuff of psychology and communication [20].

The typical approach to analyzing language involves counting word usage over pre-chosen categories of language. For example, one might place words like ‘nose’, ‘bones’, ‘hips’, ‘skin’, ‘hands’, and ‘gut’ into a *body* lexicon, and count how often words in the lexicon are used by *extraverts* or *introverts* in order to determine who talks about the body more. Of such word-category lexica, the most widely used is Linguistic Inquiry and Word Count or *LIWC*, developed over the last couple decades by human judges designating categories for common words [11], [19]. The 2007 version of *LIWC* includes 64 different categories of language ranging from part-of-speech (i.e. *articles*, *prepositions*, *past-tense verbs*, *numbers*,...) to topical categories (i.e. *family*, *cognitive mechanisms*, *affect*, *occupation*, *body*,...), as well as a few other attributes such as total number of words used [11]. Names of all 64 categories can be seen in Figure 2.

Pennebaker & King conducted one of the first extensive applications of *LIWC* to personality by examining words in a variety of domains including diaries, college writing assignments, and social psychology manuscript abstracts [21]. Their results were quite consistent across such domains, finding patterns such as *agreeable* people using more articles, *introverts* and those low in *conscientiousness* using more words signaling distinctions, and *neurotic* individuals using more negative emotion words. Mehl et al. tracks the natural speech of 96 people over two days [22]. They found similar results to Pennebaker & King and that *neurotic* and *agreeable* people tend to use more first-person singulars, people low in *openness* talk more about social processes, *extraverts* use longer words.

The recent growth of online social media has yielded great sources of personal discourse. Besides advantages due to the size of the data, the content is often personal and describes everyday concerns. Furthermore, previous research has suggested populations for online studies and Facebook are quite representative [23], [24]. Sumner et al. examined the language of 537 Facebook users with *LIWC* [25] while Holtgraves studied the text messages of 46 students [26]. Findings from these studies largely confirmed past links with LIWC but also introduced some new links such as *neurotics* using more acronyms [26] or those high in *openness* using more quotations [25].

The larger sample-sizes from social media also enabled the first study exploring personality as a function of single-word use. Yarkoni investigated LIWC categories along with single words in connection with Big-5 scores of 406 bloggers [27]. He identified single word results which would not have been caught with *LIWC*, such as ‘hug’ correlating positively with *agreeableness* (there is no physical affection category in*LIWC*), but, considering the sparse nature of words, 406 blogs does not result in comprehensive view. For example, they find only 13 significant word correlations for *conscientiousness* while we find thousands even after Bonferonni-correcting significance levels. Additionally, they did not control for age or gender although they reported roughly 75% of their subjects were female. Still, as the most thorough point of comparison for *LIWC* results with personality, Figure 2 presents the findings from Yarkoni's study along with *LIWC* results over our data.

Analogous to a personality construct, work has been done in psychology looking at the latent dimensions of self-expression. Chung and Pennebaker factor analyzed 119 adjectives used in student essays of “who you think you are” and discovered 7 latent dimensions labeled such as “sociability” or “negativity” [28]. They were able to relate these factors to the Big-5 and found only weak relations, suggesting 7 dimensions as an alternative construction. Later, Kramer and Chung ran the same method over 1000 unique words across Facebook status updates, finding three components labeled, “positive events”, “informal speech”, and “school” [29]. Although their vocabulary size was somewhat limited, we still see these as previous examples of open-vocabulary language analyses for psychology – no assumptions were made on the categories of words beyond part-of-speech.

*LIWC* has also been used extensively for studying gender and age [21]. Many studies have focused on function words (articles, prepositions, conjunctions, and pronouns), finding females use more first-person singular pronouns, males use more articles, and that older individuals use more plural pronouns and future tense verbs [30]–[32]. Other works have found males use more formal, affirmation, and informational words, while females use more social interaction, and deictic language [33]–[36]. For age, the most salient findings include older individuals using more positive emotion and less negative emotion words [30], older individuals preferring fewer self-references (i.e. ‘I’, ‘me’) [30], [31], and stylistically there is less use of negation [37]. Similar to our finding of 2000 topics (clusters of semantically-related words), Argamon et al. used factor analysis and identified 20 coherent components of word use to link gender and age, showing male components of language increase with age while female factors decrease [32].

Occasionally, studies find contradictory results. For example, multiple studies report that emoticons (i.e. ‘:)’ ‘:-(‘) are used more often by females [34], [36], [38], but Huffaker & Calvert found males use them more in a sample of 100 teenage bloggers [39]. This particular discrepancy could be sample-related – differing demographics or having a non-representative sample (Huffaker & Calvert looked at 100 bloggers, while later studies have looked at thousands of twitter users) or it could be due to differences in the domain of the text (blogs versus twitter). One should always be careful generalizing new results outside of the domain they were found as language is often dependent on context [40]. In our case we explore language in the broad context of Facebook, and do not claim our results would up under other smaller or larger contexts. As a starting point for reviewing more psychologically meaningful language findings, we refer the reader to Tauszczik & Pennebaker's 2010 survey of computerized text analysis [20].

Eisenstein et al. presented a sophisticated *open-vocabulary* language analysis of demographics [41]. Their method views language analysis as a multi-predictor to multi-output regression problem, and uses an L1 norm to select the most useful predictors (i.e. words). Part of their motivation was finding interpretable relationships between individual language features and sets of outcomes (demographics), and unlike the many predictive works we discuss in the next section, they test for significance of relationships between individual language features and outcomes. To contrast with our approach, we consider features and outcomes individually (i.e. an “L0 norm”), which we think is more ideal for our goals of explaining psychological variables (i.e. understanding openness by the words that correlate with it). For example, their method may throwout a word which is strongly predictive for only one outcome or which is collinear with other words, while we want to know all the words most-predictive for a given outcome. We also explore other types of *open-vocabulary* language features such as phrases and topics.

Similar language analyses also occurred in many fields outside of psychology or demographics [42], [43]. For example, Monroe et al. explored a variety of techniques that compare two frequencies of words – one number for each of two groups [44]. In particular, they explored frequencies across democratic versus republican speeches and settled on a Bayesian model with regularization and shrinkage based on priors of word use. Lastly, Gilbert finds words and phrases that distinguish communication up or down a power-hierarchy across 2044 Enron emails [45]. They used penalized logistic regression to fit a single model using coefficients of each feature as their “power”; this produces a good single predictive model but also means words which are highly collinear with others will be missed (we run a separate regression for each word to avoid this).

Perhaps one of the most comprehensive language analysis surveys outside of psychology is that of Grimmer & Stewart [43]. They summarize how automated methods can inexpensively allow systematic analysis and inference from large political text collections, classifying types of analyses into a of hierarchy. Additionally, they provide cautionary advice; In relation to this work, they note that dictionary methods (such as the closed-vocabulary analyses discussed here) may signal something different when used in a new domain (for example ‘crude’ may be a negative word in student essays, but be neutral in energy industry reports: ‘crude oil’). For comprehensive surveys on text analyses across fields see Grimmer & Stewart [43], O'Connor, Bamman, & Smith [42], and Tausczik & Pennebaker [46].

### Predictive Models based on Language

In contrast with the works seeking to gain *insights* about psychological variables, research focused on *predicting* outcomes have embraced data-driven approaches. Such work uses open-vocabulary linguistic features in addition to *a priori* lexicon based features in predictive models for tasks such as stylistics/authorship attribution [47]–[49], emotion prediction [50], [51], interaction or flirting detection [52], [53], or sentiment analysis [54]–[57]. In other works, ideologies of political figures (i.e. conservative to liberal) have been predicted based on language using supervised techniques [58] or unsupervised inference of ideological space [59], [60]. Sometimes these works note the highest weighted features, but with their goal being predictive accuracy, those features are not tested for significance and they usually are not the most individually distinguishing pieces of language. To elaborate, most approaches to prediction penalize the weights of words that are highly collinear with other words as they fit a single model per outcomes across all words. However, these highly collinear words which are suppressed, could have revealed important insights with an outcome. In other words, these predictive models answer the question “what is the best combination of words and weights to predict personality?” whereas we believe answering the following question is best for revealing new insights: “what words, controlled for gender and age, are individually most correlated with personality?”.

Recently, researchers have started looking at personality prediction. Early works in personality prediction used dictionary-based features such as *LIWC*. Argamon et al. (2005) noted that personality, as detected by categorical word use, was supportive for author attribution. They examined language use according to the traits of *neuroticism* and *extraversion* over approximately 2200 student essays, while focused on using function words for the prediction of gender [62]. Mairesse et al. used a variety of lexicon-based features to predict all Big-5 personality traits over approximately 2500 essays as well as 90 sets of individual spoken words [63], [64]. As a first pass at predicting personality from language in Facebook, Golbeck used *LIWC* features over a sample of 167 Facebook volunteers as well as profile information and found limited success of a regression model [65]. Similarly, Kaggle held a competition of personality prediction over Twitter messages, providing participants with language cues based on *LIWC* [66]. Results of the competition suggested personality is difficult to predict based on language in social media, but it is not clear whether such a conclusion would have been drawn had *open-vocabulary* language cues been supplied for prediction.

In the largest previous study of language and personality, Iacobelli, Gill, Nowson, and Oberlander attempted prediction of personality for 3,000 bloggers [67]. Not limited to categorical language they found open-vocabulary features, such as bigrams, to be better predictors than *LIWC* features. This motivates our exploration of open-vocabulary features for psychological insights, where we examine multi-word phrases (also called n-grams) as well as open-vocabulary category language in the form of automatically clustered groups of semantically related word (*LDA topics*, see “Linguistic Feature Extraction” in the “Materials and Methods” section). Since the application of Iacobelli et al. 's work was content customization, they focused on prediction rather than exploration of language for psychological insight. Our much larger sample size lends itself well to more comprehensive exploratory results.

Similar studies have also been undertaken for age and gender prediction in social media. Because gender and age information is more readily available, these studies tend to be larger. Argamon et al. predicted gender and age over 19,320 bloggers [32], while Burger et al. scaled up the gender prediction over 184,000 Twitter authors by using automatically guessed gender based-on gender-specific keywords in profiles. Most recently, Bamman et al. looked at gender as a function of language and social network statistics in twitter. They particularly looked at the characteristics of those whose gender was incorrectly predicted and found greater gender homophily in the social networks of such individuals [68].

**Figure 1:**
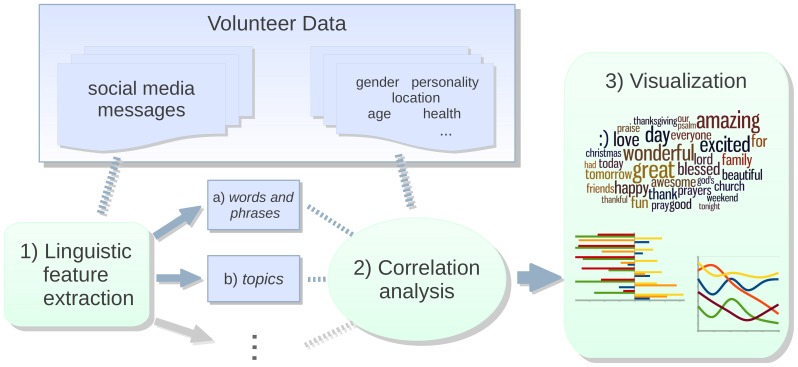
The infrastructure of our differential language analysis. 1) *Feature Extraction*. Language use features include: *(a) words and phrases*: a sequence of 1 to 3 words found using an emoticon-aware tokenizer and a collocation filter (24,530 features) *(b) topics*: automatically derived groups of words for a single topic found using the Latent Dirichlet Allocation technique [72], [75] (500 features). 2) *Correlational Analysis*. We find the correlation (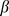 of ordinary least square linear regression) between each language feature and each demographic or psychometric outcome. All relationships presented in this work are at least significant at a Bonferroni-corrected 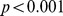 [76]. 3) *Visualization*. Graphical representation of correlational analysis output.

These past studies, mostly within the field of computer science or specifically computational linguistics, have focused on prediction for tasks such as content personalization or authorship attribution. In our work, predictive models of personality, gender, and age provide a quantitative means to compare various *open-vocabulary* sets of features with a *closed-vocabulary* set. Our primary concern is to explore the benefits of an *open-vocabulary* approach for gaining *insights*, a goal that is at least as import as prediction for psychosocial fields. Most works for gaining language-based insights in psychology are *closed-vocabulary* (for examples, see previous section), and while many works in computational linguistics are open-vocabulary, they rarely focus on insight. We introduce the term “open-vocabulary” to distinguish an approach like ours from previous approaches to gaining *insight*, and in order to encourage others seeking insights to consider similar approaches. “Differential language analysis” refers to the particular process, for which we are not aware of another name, we use in our *open-vocabulary* approach as depicted in Figure 1.

**Figure 2:**
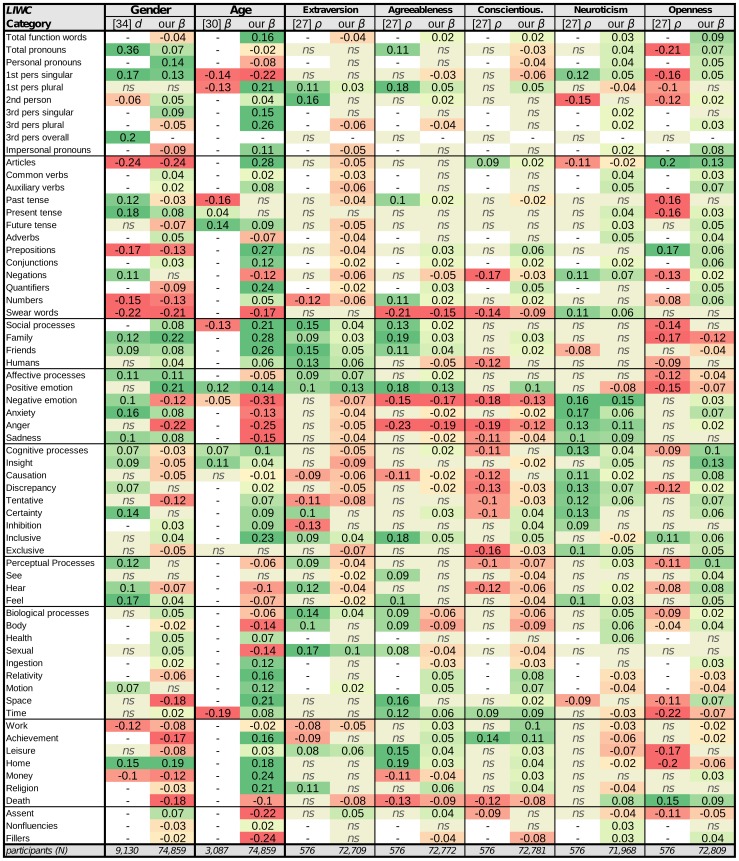
Correlation values of *LIWC* categories with gender, age, and the five factor model of personality. [34] 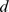: Effect size as Cohen's 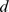 values from Newman et al. 's recent study of gender (positive is female, 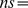 not significant at 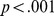) [30]. 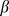: Standardized linear regression coefficients adjusted for sex, writing/talking, and experimental condition from Pennebaker and Stone's study of age (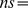 not significant at 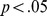) [27]. 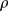: Spearman correlations values from Yarkoni's recent study of personality (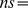 not significant at 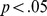). **our** 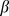: Standardized multivariate regression coefficients adjusted for gender and age for this current study over Facebook (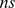 =  not significant at Bonferroni-corrected 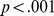).

### Contributions

**Figure 3:**
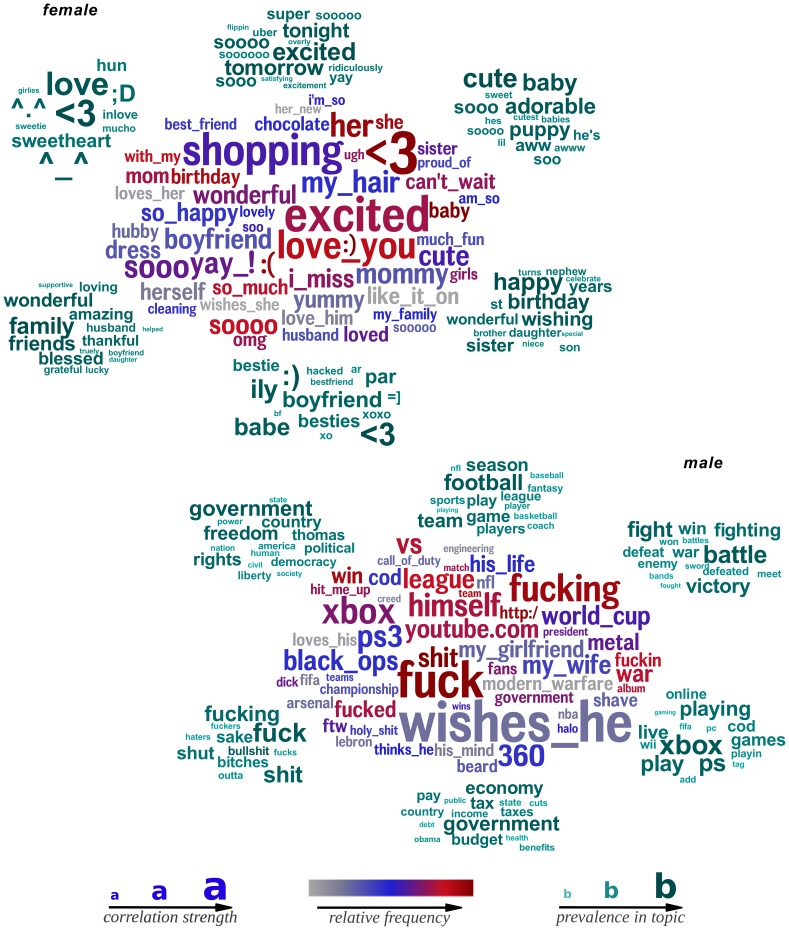
Words, phrases, and topics most highly distinguishing females and males. Female language features are shown on top while males below. Size of the word indicates the strength of the correlation; color indicates relative frequency of usage. Underscores (_) connect words of multiword phrases. *Words and phrases* are in the center; *topics*, represented as the 15 most prevalent words, surround. (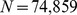: 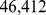 females and 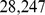 males; correlations adjusted for age; Bonferroni-corrected 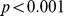).

The contributions of this paper are as follows:

- First, we present the largest study of personality and language use to date. With just under 75,000 authors, our study covers an order-of-magnitude more people and instances of language features than the next largest study ([27]). The size of our data enables qualitatively different analyses, including open vocabulary analysis, based on more comprehensive sets of language features such as *phrases* and automatically derived *topics*. Most prior studies used *a priori* language categories, presumably due in part to the sparse nature of words and their relatively small samples of people. With smaller data sets, it is difficult to find statistically significant differences in language use for anything but the most common words.
- Our *open-vocabulary* analysis yields further insights into the behavioral residue of personality types beyond those from *a priori* word-category based approaches, giving unanticipated results (correlations between language and personality, gender, or age). For example, we make the novel discoveries that mentions of an assortment of social sports and life activities (such as *basketball*, *snowboarding*, *church*, *meetings*) correlate with *emotional stability*, and that *introverts* show an interest in Japanese media (such as *anime*, *pokemon*, *manga* and Japanese emoticons: ˆ_ˆ). Our inclusion of phrases in addition to words provided further insights (e.g. that males prefer to precede ‘girlfriend’ or ‘wife’ with the possessive ‘my’ significantly more than females do for ‘boyfriend’ or ‘husband’. Such correlations provide quantitative evidence for strong links between behavior, as revealed in language use, and psychosocial variables. In turn, these results suggest undertaking studies, such as directly measuring participation in activities in order to verify the link with emotional stability.
- We demonstrate open-vocabulary features contain more information than *a priori* word-categories via their use in predictive models. We take model accuracy in out-of-sample prediction as a measure of information of the features provided to the model. Models built from words and phrases as well as those from automatically generated topics achieve significantly higher out-of-sample prediction accuracies than a standard lexica for each variable of interest (*gender*, *age*, and *personality*). Additionally, our prediction model for gender yielded state-of-the-art results for predictive models based entirely on language, yielding an out-of-sample accuracy of 91.9%.
- We present a word cloud visualization which scales words by correlation (i.e., how well they predict the given psychological variable) rather than simply scaling by frequency. Since we find thousands of significantly correlated words, visualization is key, and our *differential* word clouds provide a comprehensive view of our results (e.g. see Figure 3).
- Lastly, we offer our comprehensive *word*, *phrase*, and *topic* correlation data for future research experiments (see: wwbp.org).

## Materials and Methods

### Ethics Statement

All research procedures were approved by the University of Pennsylvania Institutional Review Board. Volunteers agreed to written informed consent.

In seeking insights from language use about personality, gender, and age, we explore two approaches. The first approach, serving as a replication of the past analyses, counts word usage over manually created *a priori* word-category lexica. The second approach, termed *DLA*, serves as out main method and is *open-vocabulary* – the words and clusters of words analyzed are determined by the data itself.

### Closed Vocabulary: Word-Category Lexica

A common method for linking language with psychological variables involves counting words belonging to manually-created categories of language. Sometimes referred to as the *word-count* approach, one counts how often words in a given category are used by an individual, the percentage of the participants' words which are from the given category:
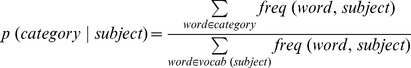
where 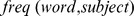 is the number of the times the participant mentions 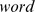 and 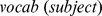 is the set of all words mentioned by the subject.

We use ordinary least squares regression to link word categories with author attributes, fitting a linear function between explanatory variables (*LIWC* categories) and dependent variables (such as a trait of personality, e.g. extraversion). The coefficient of the target explanatory variable (often referred to as 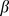) is taken as the strength of relationship. Including other variables allows us to adjust for covariates such as gender and age to provide the unique effect of a given language feature on each psychosocial variable.

### Open Vocabulary: Differential Language Analysis

Our technique, *differential language analysis* (*DLA*), is based on three key characteristics. It is

1. *Open-vocabulary* – it is not limited to predefined word lists. Rather, linguistic features including words, phrases, and topics (sets of semantically related words) are automatically determined from the texts. (I.e., it is “data-driven”.) This means *DLA* is classified as a type of open-vocabulary approach.
2. *Discriminating* – it finds key linguistic features that distinguish psychological and demographic attributes, using stringent significance tests.
3. *Simple* – it uses simple, fast, and readily accepted statistical techniques.

We depict the components of this approach in Figure 1, and describe the three steps: 1) linguistic feature extraction, 2) correlational analysis, and 3) visualization in the following sections.

#### 1. Linguistic Feature Extraction

We examined two types of linguistic features: a) *words and phrases*, and b) *topics*. *Words and phrases* consisted of sequences of 1 to 3 words (often referred to as ‘n-grams’ of size 1 to 3). What constitutes a word is determined using a tokenizer, which splits sentences into tokens (“words”). We built an emoticon-aware tokenizer on top of Pott's “happyfuntokenizer” allowing us to capture emoticons like ‘
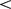
3’(a heart) or ‘:-)’ (a smile), which most tokenizers incorrectly divide up as separate pieces of punctuation. When extracting phrases, we keep only those sequences of words with high informative value according to pointwise mutual information (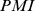) [69], [70], a ratio of the joint-probability to the independent probability of observing the phrase:
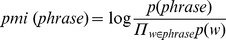



In practice, we kept phrases with 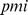 values greater than 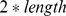, where 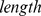 is the number of words contained in the phrase, ensuring that phrases we do keep are informative parts of speech and not just accidental juxtapositions. All word and phrase counts are normalized by each subject's total word use (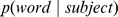), and we apply the Anscombe transformation [71] to the normalized values for variance stabilization (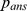):
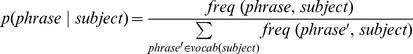


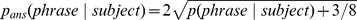
where 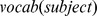 returns a list of all words and phrases used by that subject. These Anscombe transformed “relative frequencies” of words or phrases (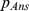) are then used as the independent variables in all our analyses. Lastly, we restrict our analysis to those words and phrases which are used by at least 1% of our subjects, keeping the focus on common language.

The second type of linguistic feature, *topics*, consists of word clusters created using Latent Dirichlet Allocation (LDA) [72], [73]. The LDA generative model assumes that documents (i.e. Facebook messages) contain a combination of topics, and that topics are a distribution of words; since the words in a document are known, the latent variable of topics can be estimated through Gibbs sampling [74]. We use an implementation of the LDA algorithm provided by the Mallet package [75], adjusting one parameter (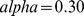) to favor fewer topics per document, since individual Facebook status updates tend to contain fewer topics than the typical documents (newspaper or encyclopedia articles) to which LDA is applied. All other parameters were kept at their default. An example of such a model is the following sets of words (*tuesday, monday, wednesday, friday, thursday, week, sunday, saturday*) which clusters together days of the week purely by exploiting their similar distributional properties across messages. We produced the 2000 topics shown in Table S1 as well as on our website.

To use topics as features, we find the probability of a subject's use of each 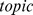:
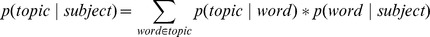
where 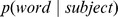 is the normalized word use by that subject and 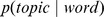 is the probability of the topic given the word (a value provided from the LDA procedure). The prevalence of a word in a topic is given by 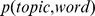, and is used to order the words within a topic when displayed.

#### 2. Correlational Analysis

Similar to word categories, distinguishing open-vocabulary words, phrases, and topics can be identified using ordinary least squares regression. We again take the coefficient of the target explanatory variable as its correlation strength, and we include other variables (e.g. age and gender) as covariates to get the unique effect of the target explanatory variable. Since we explore many features at once, we consider coefficients significant if they are less than a Bonferroni-corrected [76] two-tailed 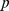 of 0.001. (I.e., when examining 20,000 features, a passing p-value is less than 0.001 divided by 20,000 which is 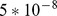).

Our correlational analysis produces a comprehensive list of the most distinguishing language features for any given attribute, *words, phrases,* or *topics* which maximally discriminate a given target variables. For example, when we correlate the target variables geographic elevation with language features (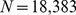, 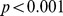, adjusted for gender and age), we find ‘beach’ the most distinguishing feature for low elevation localities, and ‘the mountains’ to be among the most distinguishing features for high elevation localities, (i.e., people in low elevations talk about the beach more, whereas people at high elevations talk about the mountains more). Similarly, we find the most distinguishing topics to be *(beach, sand, sun, water, waves, ocean, surf, sea, toes, sandy, surfing, beaches, sunset, Florida, Virginia)* for low elevations and *(Colorado, heading, headed, leaving, Denver, Kansas, City, Springs, Oklahoma, trip, moving, Iowa, KC, Utah, bound)* for high elevations. Others have looked at geographic location [77].

#### 3. Visualization

An analysis over tens of thousands of language features and multiple dimensions results in hundreds of thousands of statistically significant correlations. Visualization is thus critical for their interpretation. We use word clouds [78] to intuitively summarize our results. Unlike most word clouds, which scale word size by their frequency, we scale word size according to the strength of the correlation of the word with the demographic or psychological measurement of interest, and we use color to represent frequency over all subjects; that is, larger words indicate stronger correlations, and darker colors indicate more frequently used words. This provides a clear picture of which words and phrases are most discriminating while not losing track of which ones are the most frequent. Word clouds scaled by frequency are often used to summarize news, a practice that has been critiqued for inaccurately representing articles [79]. Here, we believe the word cloud is an appropriate visualization because the individual words and phrases we depict in it are the actual results we wish to summarize. Further, scaling by correlation coefficient rather than frequency gives clouds that distinguish a given outcome.

Word clouds can also used to represent distinguishing topics. In this case, the size of the word within the topic represents its prevalence among the cluster of words making up the topic. We use the 6 most distinguishing topics and place them on the perimeter of the word clouds for *words and phrases*. This way, a single figure gives a comprehensive view of the most distinguishing words, phrases, and topics for any given variables of interest. See Figure 3 for an example.

To reduce the redundancy of results, we automatically prune language features containing information already provided by a feature with higher correlation. First, we sort language features in order of their correlation with a target variable (such as a personality trait). Then, for phrases, we use frequency as a proxy for informative value [80], and only include additional phrases if they contain more informative words than previously included phrases with matching words. For example, consider the phrases ‘day’, ‘beautiful day’, and ‘the day’, listed in order of correlation from greatest to least; ‘Beautiful day’ would be kept, because ‘beautiful’ is less frequent than ‘day’ (i.e., it is adding informative value), while ‘the day’ would be dropped because ‘the’ is more frequent than ‘day’ (thus it is not contributing more information than we get from ‘day’). We do a similar pruning for topics: A lower-ranking topic is not displayed if more than 25% of its top 15 words are also contained in the top 15 words of a higher ranking topic. These discarded relationships are still statistically significant, but removing them provides more room in the visualizations for other significant results, making the visualization as a whole more meaningful.

Word clouds allow one to easily view the features most correlated with polar outcomes; we use other visualizations to display the variation of correlation of language features with continuous or ordinal dependent variables such as age. A standard time-series plot works well, where the horizontal axis is the dependent variable and the vertical axis represents the standard score of the values produced from feature extraction. When plotting language as a function of age, we fit first-order LOESS regression lines [81] to the age as the x-axis data and standardized frequency as the y-axis data over all users. We are able to adjust for gender in the regression model by including it as a covariate when training the LOESS model and then using a neutral gender value when plotting.

### Data Set: Facebook Status Updates

Our complete dataset consists of approximately 19 million Facebook status updates written by 136,000 participants. Participants volunteered to share their status updates as part of the *My Personality* application, where they also took a variety of questionnaires [12]. We restrict our analysis to those Facebook users meeting certain criteria: They must indicate English as a primary language, have written at least 1,000 words in their status updates, be less than 65 years (to avoid the non-representative sample above 65), and indicate both gender and age (for use as controls). This resulted in 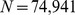 volunteers, writing a total of 309 million words (700 million feature instances of words, phrases, and topics) across 15.4 million status updates. From this sample each person wrote an average of 4,129 words over 206 status updates, and thus 20 words per update. Depending on the target variable, this number slightly varies as indicated in the caption of each result.

The personality scores are based on the International Personality Item Pool proxy for the NEO Personality Inventory Revised (NEO-PI-R) [14], [82]. Participants could take 20 to 100 item versions of the questionnaire, with a retest reliability of 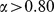 [12]. With the addition of gender and age variables, this resulted in seven total dependent variables studied in this work, which are depicted in Table 1 along with summary statistics. Personality distributions are quite typical with means near zero and standard deviations near 1. The statuses ranged over 34 months, from January 2009 through October 2011. Previously, profile information (i.e. network metrics, relationship status) from users in this dataset have been linked with personality [83], but this is the first use of its status updates.

**Table 1:** Summary statistics for gender, age, and the five factor model of personality.

	*N*	*mean*	*standard deviation*	*skewness*
**Gender**	74859	0.62	0.49	−0.50
**Age**	74859	23.43	8.96	1.77
**Extraversion**	72709	−0.07	1.01	−0.34
**Agreeableness**	72772	0.03	1.00	−0.40
**Conscientiousness**	72781	−0.04	1.01	−0.09
**Neuroticism**	71968	0.14	1.04	−0.21
**Openness**	72809	0.12	0.97	−0.48

These represent the seven dependent variables studied in this work. Gender ranged from 0 (male) to 1(female). Age ranged from 13 to 65. Personality questionnaires produce values along a standardized continuum.

## Results

Results of our analyses over gender, age, and personality are presented below. As a baseline, we first replicate the commonly used *LIWC* analysis on our data set. We then present our main results, the output of our method, *DLA*. Lastly, we explore empirical evidence that *open-vocabulary* features provide more information than those from an *a priori* lexicon through use in a predictive model.

### Closed Vocabulary

Figure 2 shows the results of applying the *LIWC* lexicon to our dataset, along side-by-side with the most comprehensive previous studies we could find for *gender*, *age*. and *personality* [27], [30], [34]. In our case, correlation results are 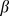 values from an ordinary least squares linear regression where we can adjust for gender and age to give the unique effect of the target variable. One should keep in mind that it is often found that effect sizes tend to be relatively smaller as sample sizes increase and become more stable [84].

Even though the previous studies listed did not look at Facebook, a majority of the correlations we find agree in direction. Some of the largest correlations emerge for the LIWC *articles* category, which consists of determiners like ‘the’, 'a’, ‘an’ and serves as a proxy for the use of more nouns. Articles are highly predictive of males, being older, and *openness*. As a content-related language variable, the *anger* category also proved highly predictive for *males* as well as younger individuals, those low in *agreeableness* and *conscientiousness*, and high in *neuroticism*. *Openness* had the least agreement with the comparison study; roughly half of our results were in the opposite direction from the prior work. This is not too surprising since *openness* exhibits the most variation across conditions of other studies (for examples, see [25], [27], [65]), and its component traits are most loosely related [85].

### Open Vocabulary

Our *DLA* method identifies the most distinguishing language features (*words, phrases*: a sequence of 1 to 3 words, or *topics*: a cluster of semantically related words) for any given attribute. Results progress from a one variable proof of concept (gender), to the multiple variables representing age groups, and finally to all 5 dimensions of personality.

#### Language of Gender

Gender provides a familiar and easy to understand proof of concept for open-vocabulary analysis. Figure 3 presents word clouds from age-adjusted gender correlations. We scale word size according to the strength of the relation and we use color to represent overall frequency; that is, larger words indicate stronger correlations, and darker colors indicate frequently used words. For the *topics*, groups of semantically-related words, the size indicate the relative prevalence of the word within the cluster as defined in the methods section. All results are significant at Bonferroni-corrected [76] 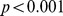.

Many strong results emerging from our analysis align with our *LIWC* results and past studies of gender. For example, females used more emotion words [86], [87] (e.g., ‘excited’), and first-person singulars [88], and they mention more psychological and social processes [34] (e.g., ‘love you’ and ‘
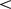
3’ –a heart). Males used more swear words, object references (e.g., ‘xbox’ and swear words) [34], [89].

Other results of ours contradicted past studies, which were based upon significantly smaller sample sizes than ours. For example, in 100 bloggers Huffaker et al. [39] found males use more emoticons than females. We calculated power analyses to determine the sample size needed to confidently find such significant results. Since the Bonferonni-correction we use elsewhere in this work is overly stringent (i.e. makes it harder than necessary to pass significance tests), for this result we applied the Benjamini-Hochberg false discovery rate procedure for multiple hypothesis testing [90]. Rerunning our language of gender analysis on reduced random samples of our subjects resulted in the following number of significant correlations (Benjamini-Hochberg tested 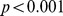): 50 subjects: 0 significant correlations, 500 subjects: 7 correlations; 5,000 subjects: 1,489 correlations; 50,000 subjects: 13,152 correlations (more detailed results of power analyses across gender, age, and personality can be found in Figure S1). Thus, traditional study sample sizes, which are closer to 50 or 500, are not powerful enough to do data-driven DLA over individual words.

One might also draw insights based on the gender results. For example, we noticed ‘my wife’ and ‘my girlfriend’ emerged as strongly correlated in the male results, while simply ‘husband’ and ‘boyfriend’ were most predictive for females. Investigating the frequency data revealed that males did in fact precede such references to their opposite-sex partner with ‘my’ significantly more often than females. On the other hand, females were more likely to precede ‘husband’ or ‘boyfriend’ with ‘her’ or ‘amazing’ and a greater variety of words, which is why ‘my husband’ was not more predictive than ‘husband’ alone. Furthermore, this suggests the male preference for the possessive ‘my’ is at least partially due to a lack of talking about others' partners.

#### Language of Age

**Figure 4:**
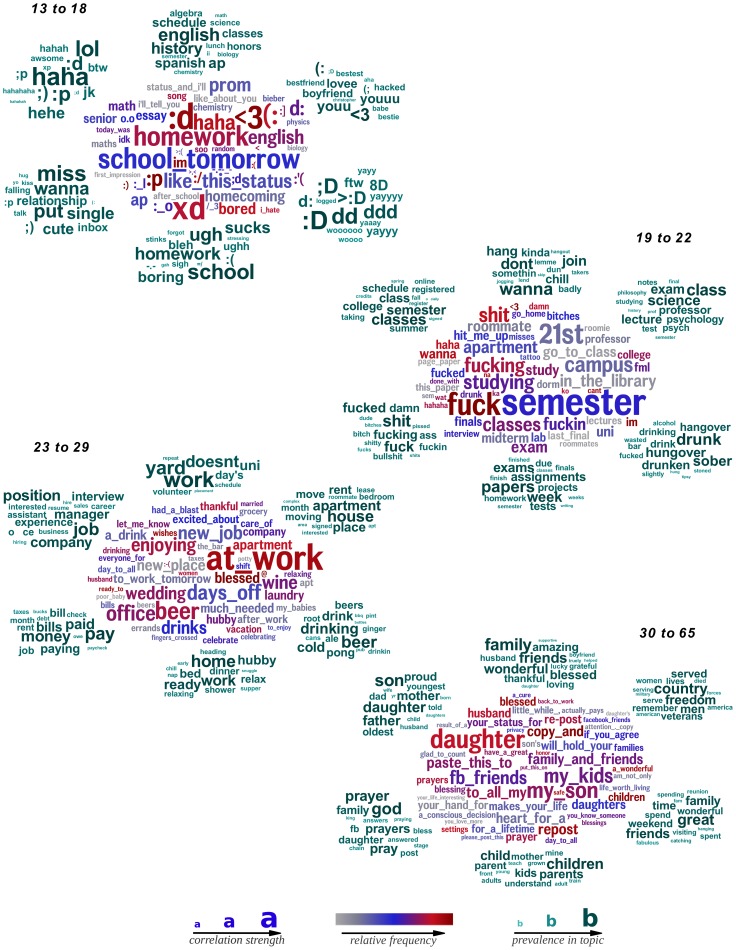
Words, phrases, and topics most distinguishing subjects aged *13 to 18*, *19 to 22*, *23 to 29*, and *30 to 65*. Ordered from top to bottom: *13 to 18 19 to 22 23 to 29*, and *30 to 65*. *Words and phrases* are in the center; *topics*, represented as the 15 most prevalent words, surround. (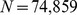; correlations adjusted for gender; Bonferroni-corrected 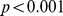).

Figure 4 shows the word cloud (center) and most discriminating topics (surrounding) for four age buckets chosen with regard to the distribution of ages in our sample (Facebook has many more young people). We see clear distinctions, such as use of slang, emoticons, and Internet speak in the youngest group (e.g. ':)’, ‘idk’, and a couple *Internet speak* topics) or work appearing in the 23 to 29 age group (e.g. ‘at work’, ‘new job’, as a *job position* topic). We also find subtle changes of topics progressing from one age group to the next. For example, we see a *school* related topic for 13 to 18 year olds (e.g. ‘school’, ‘homework’, ‘ugh’), while we see a *college* related topic for 19 to 22 year olds (e.g. ‘semester’, ‘college’, ‘register’). Additionally, consider the *drunk* topic (e.g. ‘drunk’, ‘hangover’, ‘wasted’) that appears for 19 to 22 year olds and a more reserved *beer* topic (e.g. ‘beer’, ‘drinking’, ‘ale’) for 23 to 29 year olds.

**Figure 5:**
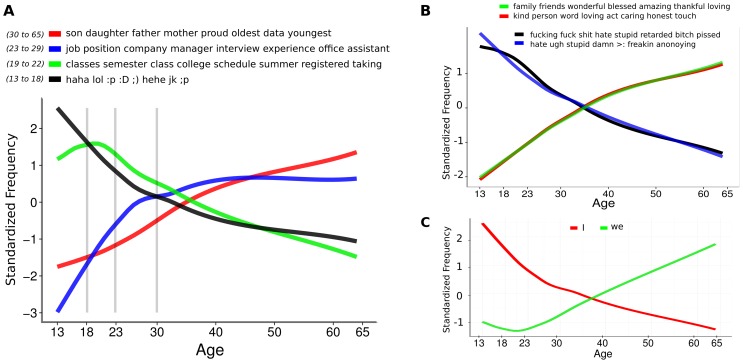
Standardized frequency of topics and words across age. **A**. Standardized frequency for the best topic for each of the 4 age groups. Grey vertical lines divide groups: 13 to 18 (black: 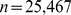 out of 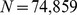), 19 to 22 (green: 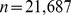), 23 to 29 (blue: 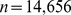), and 30+ (red: 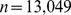). Lines are fit from first-order LOESS regression [81] controlled for gender. **B**. Standardized frequency of social topic use across age. **C**. Standardized ‘I’, ‘we’ frequencies across age.

In general, we find a progression of school, college, work, and family when looking at the predominant topics across all age groups. *DLA* may be valuable for the generation of hypotheses about life span developmental age differences. Figure 5A shows the relative frequency of the most discriminating topic for each age group as a function of age. Typical concerns peak at different ages, with the topic concerning relationships (e.g. ‘son’, ‘daughter’, ‘father’, ‘mother’) continuously increasing across life span. On a similar note, Figure 5C shows ‘we’ increases approximately linearly after the age of 22, whereas ‘I’ monotonically decreases. We take this as a proxy for social integration [19], suggesting the increasing importance of friendships and relationships as people age. Figure 5B reinforces this hypothesis by presenting a similar pattern based on other social topics. One limitation of our dataset is the rarity of older individuals using social media; we look forward to a time in which we can track fine-grained language differences across the entire lifespan.

#### Language of Personality

We created age and gender-adjusted word clouds for each personality factor based on around 72 thousand participants with at least 1,000 words across their Facebook status updates, who took a Big Five questionnaire [91].

**Figure 6:**
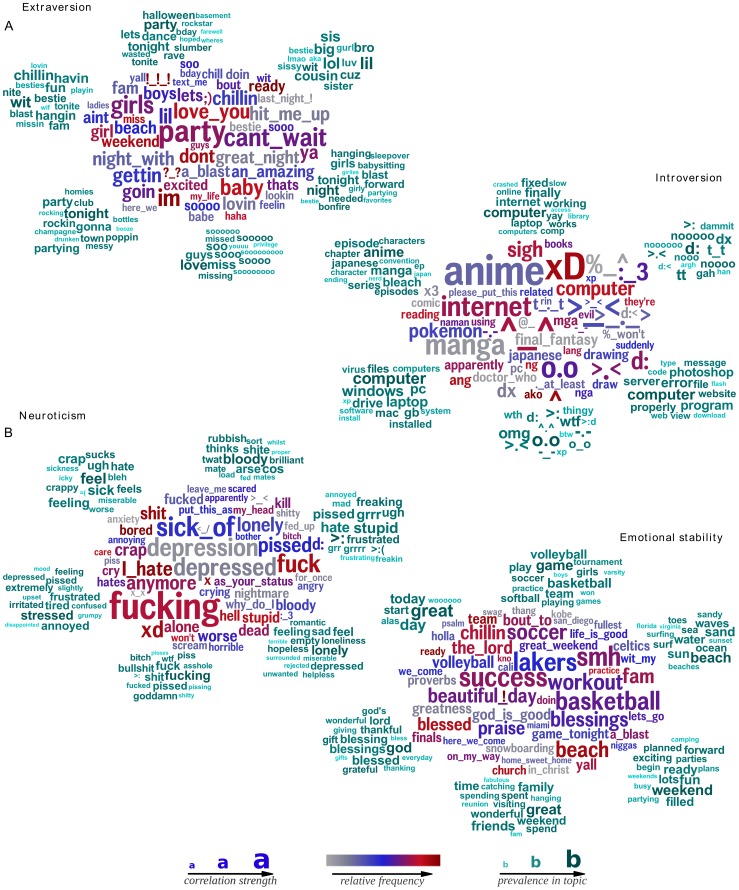
Words, phrases, and topics most distinguishing *extraversion* from *introversion* and *neuroticism* from *emotional stability*. **A**. Language of extraversion (left, e.g., ‘party’) and introversion (right, e.g., ‘computer’); 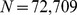. **B**. Language distinguishing neuroticism (left, e.g. ‘hate’) from emotional stability (right, e.g., ‘blessed’); 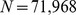 (adjusted for age and gender, Bonferroni-corrected 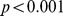). Figure S8 contains results for *openness*, *conscientiousness*, and *agreeableness*.

Figure 6 shows word clouds for extraversion and neuroticism. (See Figure S2 for openness, conscientiousness, and agreeableness.) The dominant words in each cluster were consistent with prior lexical and questionnaire work [14]. For example, extraverts were more likely to mention social words such as ‘party’, ‘love you’, ‘boys’, and ‘ladies’, whereas introverts were more likely to mention words related to solitary activities such as ‘computer’, ‘Internet’, and ‘reading’. In the openness cloud, words such as ‘music’, ‘art’, and ‘writing’ (i.e., creativity), and ‘dream’, ‘universe’, and ‘soul’ (i.e., imagination) were discriminating [85].

Topics were also found reflecting similar concepts as the words, some of which would not have been captured with *LIWC*. For example, although *LIWC* has socially related categories, it does not contain a *party* topic, which emerges as a key distinguishing feature for extraverts. Topics related to other types of social events are listed elsewhere, such as a sports topic for low neuroticism (emotional stability). Additionally, Figure 6 shows the advantage of having phrases in the analysis to get clearer signal: e.g. people high in neuroticism mentioned ‘sick of’, and not just ‘sick’.

While many of our results confirm previous research, demonstrating the instrument's face validity, our word clouds also suggest new hypotheses. For example, Figure 6 (bottom-right) shows language related to emotional stability (low neuroticism). Emotionally stable individuals wrote about enjoyable social activities that may foster greater emotional stability, such as ‘sports’, ‘vacation’, ‘beach’, ‘church’, ‘team’, and a *family time* topic. Additionally, results suggest that introverts are interested in Japanese media (e.g. ‘anime’, ‘manga’, ‘japanese’, Japanese style emoticons: ˆ_ˆ, and an *anime* topic) and that those low in *openness* drive the use of shorthands in social media (e.g. ‘2day’, ‘ur’, ‘every 1’). Although these are only language correlations, they show how *open-vocabulary* analyses can illuminate areas to explore further.

### Predictive Evaluation

Here we present a quantitative evaluation of open-vocabulary and closed vocabulary language features. Although we have thus far presented subjective evidence that open-vocabulary features contribute more information, we hypothesize empirically that the inclusion of open-vocabulary features leads to prediction accuracies above and beyond that of closed-vocabulary. We randomly sampled 25% of our participants as test data, and used the remaining 75% as training data to build our predictive models.

We use a linear support vector machine (*SVM*) [92] for classifying the binary variable of gender, and ridge regression [93] for predicting age and each factor of personality. Features were first run through principal component analysis to reduce the feature dimension to half of the number of users. Both SVM classification and ridge regression utilize a regularization parameter, which we set by validation over the training set (we defined a small validation set of 10% of the training set which we tested various regularization parameters over while fitting the model to the other 90% of the training set in order to select the best parameter). Thus, the predictive model is created without any outcome information outside of the training data, making the test data an out-of-sample evaluation.

As open-vocabulary features, we use the same units of language as *DLA*: *words and phrases* (n-grams of size 1 to 3, passing a collocation filter) and *topics*. These features are outlined precisely under the “Linguistic Feature Extraction” section presented earlier. As explained in that section, we use Anscombe transformed relative frequencies of *words and phrases* and the conditional probability of a *topic* given a subject. For closed vocabulary features, we use the *LIWC* categories of language calculated as the relative frequency of a user mentioning a word in the category given their total word usage. We do not provide our models with anything other than these language usage features (independent variables) for prediction, and we use usage of all features (not just those passing significance tests from *DLA*).

As shown in Table 2, we see that models created with *open vocabulary* features significantly (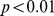) outperformed those created based on *LIWC* features. The *topics* results are of particular interest, because these automatically clustered word-category lexica were not created with any human or psychological data – only knowing what words occurred in messages together. Furthermore, we see that a model which includes *LIWC* features on top of the *open-vocabulary words*, *phrases*, and *topics* does not result in any improvement suggesting that the open-vocabulary features are able to capture predictive information which fully supersedes *LIWC*.

**Table 2:** Comparison of *LIWC* and *open-vocabulary* features within predictive models of gender, age, and personality.

	Gender	Age	Extraversion	Agreeableness	Conscientious.	Neuroticism	Openness
features	*accuracy*	*R*	*R*	*R*	*R*	*R*	*R*
*LIWC*	78.4%	.65	.27	.25	.29	.21	.29
*Topics*	**87.5%**	**.80**	**.32**	**.29**	**.33**	**.28**	**.38**
*WordPhrases*	**91.4%**	**.83**	**.37**	**.29**	**.34**	**.29**	**.41**
*WordPhrases + Topics*	**91.9%**	**.84**	**.38**	**.31**	**.35**	**.31**	**.42**
*Topics + LIWC*	**89.2%**	**.80**	**.33**	**.29**	**.33**	**.28**	**.38**
*WordPhrases + LIWC*	**91.6%**	**.83**	**.38**	**.30**	**.34**	**.30**	**.41**
*WordPhrases + Topics + LIWC*	**91.9%**	**.84**	**.38**	**.31**	**.35**	**.31**	**.42**

*accuracy*: percent predicted correctly (for discrete binary outcomes). *R*: Square-root of the coefficient of determination (for sequential/continuous outcomes). *LIWC*: *A priori* word-categories from Linguistic Inquiry and Word Count. *Topics*: Automatically created *LDA* topic clusters. *WordPhrases*: words and phrases (n-grams of size 1 to 3 passing a collocation filter). Bold indicates significant (p<.01) improvement over the baseline set of features (use of *LIWC* alone).

For personality we saw the largest relative improvement between *open-vocabulary* approaches and *LIWC*. Our best personality 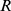 score of 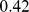 fell just above the standard “correlational upper-limit” for behavior to predict personality (a Pearson correlation of 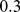 to 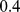) [94], [95]. Some researchers have discretized the personality scores for prediction, and classified people as being high or low (one standard deviation above or below the mean or top and bottom quartiles, throwing out the middle) in each trait [61], [64], [67]. When we do such an approach, our scores are in similar ranges to such literature: 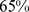 to 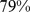 classification accuracy. Of course, such a high/low model cannot directly be used for classifying unlabeled people as one would also need to know who fits in the middle. Regression is a more appropriate predictive task for continuous outcomes like age and personality, even though 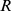 scores are naturally smaller than binary classification accuracies.

We ran an additional tests to evaluate only those words and phrases, topics, or *LIWC* categories that are selected via differential language analysis rather than all features. Thus, we used only those language features that significantly correlated (Bonferonni-corrected 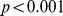) with the outcome being predicting. To keep consistent with the main evaluation, we used no controls, and so one could view this as a univariate feature selection over each type of feature independently. We again found significant improvement from using the open-vocabulary features over *LIWC* and no significant changes in accuracy overall. These results are presented in Table S2.

In addition to demonstrating the greater informative value of *open-vocabulary* features, we found our results to be state-of-the-art. The highest previous *out-of-sample* accuracies for gender prediction based *entirely* on language were 88.0% over twitter data [68] while our classifiers reach an accuracy of *91.9%*. Our increased performance could be attributed to our set of language features, a strong predictive algorithm (the support vector machine), and the large sample of Facebook data.

## Discussion

Online social media such as Facebook are a particularly promising resource for the study of people, as “status” updates are self-descriptive, personal, and have emotional content [7]. Language use is objective and quantifiable behavioral data [96], and unlike surveys and questionnaires, Facebook language allows researchers to observe individuals as they freely present themselves in their own words. *Differential language analysis* (*DLA*) in social media is an unobtrusive and non-reactive window into the social and psychological characteristics of people's everyday concerns.

Most studies linking language with psychological variables rely on *a priori* fixed sets of words, such as the *LIWC* categories carefully constructed over 20 years of human research [11]. Here, we show the benefits of an *open-vocabulary* approach in which the words analyzed are based on the data itself. We extracted *words*, *phrases*, and *topics* (automatically clustered sets of words) from millions of Facebook messages and found the language that correlates most with gender, age, and five factors of personality. We discovered insights not found previously and achieved higher accuracies than *LIWC* when using our *open-vocabulary* features in a predictive model, achieving state-of-the-art accuracy in the case of gender prediction.

Exploratory analyses like *DLA* change the process from that of testing theories with observations to that of data-driven identification of new connections [97],[98]. Our intention here is not a complete replacement for *closed-vocabulary* analyses like *LIWC*. When one has a specific theory in mind or a small sample size, an *a priori* list of words can be ideal; in an open-vocabulary approach, the concept one cares about can be drowned out by more predictive concepts. Further, it may be easier to compare static *a priori* categories of words across studies. However, automatically clustering words into coherent topics allows one to potentially discover categories that might not have been anticipated (e.g. sports teams, kinds of outdoor exercise, or Japanese cartoons). Open-vocabulary approaches also save labor in creating categories. They consider all words encountered and thus are able to adapt well to the evolving language in social media or other genres. They are also transparent in that the exact words driving correlations are not hidden behind a level of abstraction. Given lots of text and dependent variables, an open-vocabulary approach like *DLA* can be immediately useful for many areas of study; for example, an economist contrasting sport utility with hybrid vehicle drivers, a political scientist comparing democrats and republicans, or a cardiologist differentiating people with positive versus negative outcomes of heart disease.

Like most studies in the social sciences, this work is still subject to sampling and social desirability biases. Language connections with psychosocial variables are often dependent on context [40]. Here, we examined language in a large sample of the broad context of Facebook. Under different contexts, it is likely some results would differ. Still, the sample sizes and availability of demographic information afforded by social media bring us closer to a more ideal representative sample [99]. Our current results have face validity (subjects in high elevations talk about ‘the mountains’), tie in with other research (neurotic people disproportionately use the phrase ‘depressed’), suggest new hypotheses (an active life implies emotional stability), and give detailed insights (males prefer to precede ‘wife’ with the possessive ‘my’ more so than females precede ‘husband’ with ‘my’).

Over the past one-hundred years, surveys and questionnaires have illuminated our understanding of people. We suggest that new multipurpose instruments such as *DLA* emerging from the field of computational social science shed new light on psychosocial phenomena.

## Supporting Information

Figure S1
**Power analyses for all outcomes examined in this work.** Number of features passing a Benjamini-Hochberg false-discovery rate of 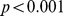 as a function of the number of users sampled, out of the maximum 24,530 words and phrases used by at least 1% of users.(TIF)

Figure S2
**Words, phrases, and topics most distinguishing** ***agreeableness*** **,** ***conscientiousness*** **, and** ***openness*** **.** A. Language of high agreeableness (left) and low agreeableness (right); 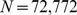. B. Language of high conscientiousness (left) and low conscientiousness (right); 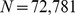. C. Language of openness (left) and closed to experience (right); 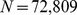 (adjusted for gender and age, Bonferroni-corrected 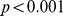).(TIF)

Table S1 **The 15 most prevalent words for the 2000 automatically generated** ***topics*** **used in our study.** All topics available here: wwbp.org/public_data/2000topics.top20freqs.keys.csv.
(XLS)

Table S2 **Prediction results when selecting features via differential language analysis.** *accuracy*: percent predicted correctly (for discrete binary outcomes). *R*: Square-root of the coefficient of determination (for sequential/continuous outcomes). *LIWC*: *A priori* word-categories from Linguistic Inquiry and Word Count. *Topics*: Automatically created *LDA* topic clusters. *WordPhrases*: words and phrases (n-grams of size 1 to 3 passing a collocation filter). Bold indicates significant (*P*<.01) improvement over the baseline set of features (use of *LIWC* alone). Differential language analysis was run over the training set, and only those features significant at Bonferonni-corrected *P*<0.001 were included during training and testing. No controls were used so as to be consistent with the evaluation in the main paper, and so one could consider this a univariate feature selection. On average results are just below those of not using *differential language analysis* to select features but there is no significant difference.
(PDF)
