# Supplementary figures and images for: Personality, Gender, and Age in the Language of Social Media: The Open-Vocabulary Approach
Source: PLoS One. 2013 Sep 25;8(9):e73791. doi: 10.1371/journal.pone.0073791 (PMC3783449; doi:10.1371/journal.pone.0073791)

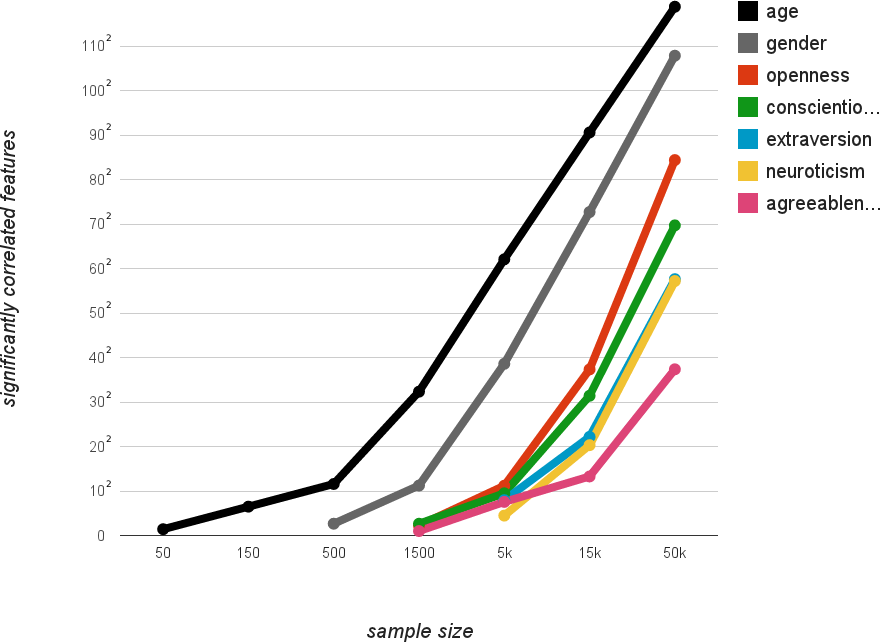

Supplement: Figure S1 — Power analyses for all outcomes examined in this work. Number of features passing a Benjamini-Hochberg false-discovery rate of as a function of the number of users sampled, out of the maximum 24,530 words and phrases used by at least 1% of users. (TIF) [file pone.0073791.s001.tif]

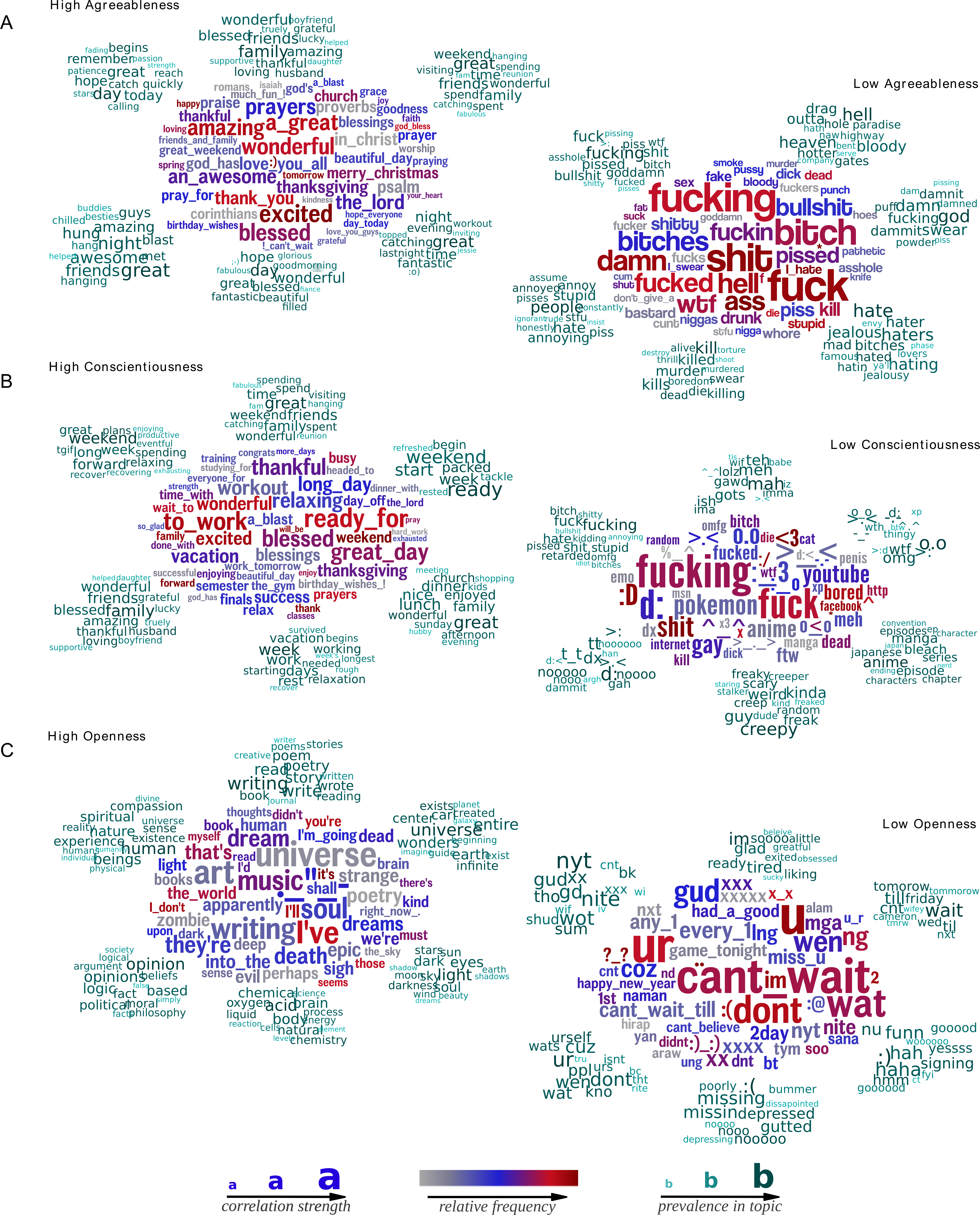

Supplement: Figure S2 — Words, phrases, and topics most distinguishing agreeableness , conscientiousness , and openness . A. Language of high agreeableness (left) and low agreeableness (right); . B. Language of high conscientiousness (left) and low conscientiousness (right); . C. Language of openness (left) and closed to experience (right); (adjusted for gender and age, Bonferroni-corrected ). (TIF) [file pone.0073791.s002.tif]
