# Supplementary material for: Personality, Gender, and Age in the Language of Social Media: The Open-Vocabulary Approach
Source: PLoS One. 2013 Sep 25;8(9):e73791. doi: 10.1371/journal.pone.0073791 (PMC3783449; doi:10.1371/journal.pone.0073791)
Supplement: Table S2 — Prediction results when selecting features via differential language analysis. accuracy: percent predicted correctly (for discrete binary outcomes). R: Square-root of the coefficient of determination (for sequential/continuous outcomes). LIWC: A priori word-categories from Linguistic Inquiry and Word Count. Topics: Automatically created LDA topic clusters. WordPhrases: words and phrases (n-grams of size 1 to 3 passing a collocation filter). Bold indicates significant (P<.01) improvement over the baseline set of features (use of LIWC alone). Differential language analysis was run over the training set, and only those features significant at Bonferonni-corrected P<0.001 were included during training and testing. No controls were used so as to be consistent with the evaluation in the main paper, and so one could consider this a univariate feature selection. On average results are just below those of not using differential language analysis to select features but there is no significant difference. (PDF) [file pone.0073791.s004.pdf]

Supporting Table 2. Prediction results when selecting features via differential language analysis.

features	**Gender** *accuracy*	**Age** *R*	**Extraversion** *R*	**Agreeableness** *R*	**Conscientious.** *R*	**Neuroticism** *R*	**Openness** *R*
LIWC	77.7%	.65	.25	.25	.29	.22	.28
Topics	**88.2%**	**.79**	**.34**	**.28**	**.34**	**.28**	**.39**
WordPhrases	**91.8%**	**.81**	**.37**	**.27**	**.34**	**.28**	**.40**
WordPhrases + Topics	**92.0%**	**.82**	**.38**	**.29**	**.35**	**.30**	**.41**
Topics + LIWC	**89.2%**	**.80**	**.35**	**.28**	**.34**	**.28**	**.40**
WordPhrases + LIWC	**91.8%**	**.81**	**.38**	**.28**	**.34**	**.29**	**.40**
WordPhrases + Topics + LIWC	**92.0%**	**.82**	**.38**	**.30**	**.35**	**.30**	**.41**

accuracy: percent predicted correctly (for discrete binary outcomes). *R*: Square-root of the coefficient of determination (for sequential / continuous outcomes). *LIWC*: *A priori* word-categories from Linguistic Inquiry and Word Count. *Topics*: Automatically created *LDA* topic clusters. *WordPhrases*: words and phrases (n-grams of size 1 to 3 passing a collocation filter). Bold indicates significant ($p < .01$) improvement over the baseline set of features (use of *LIWC* alone). Differential language analysis was run over the training set, and only those features significant at Bonferonni-corrected $p < 0.001$ were included during training and testing. No controls were used so as to be consistent with the evaluation in the main paper, and so one could consider this a univariate feature selection. On average results are just below those of not using *differential language analysis* to select features but there is no significant difference.
